# 1792. SARS-CoV-2 Genomic Surveillance System Development as a Model for Other Emerging Pathogens in Texas

**DOI:** 10.1093/ofid/ofad500.1621

**Published:** 2023-11-27

**Authors:** Saroj Rai, Rania Milleron, Rachel Lee, Blake M Hanson, Marlisa Hardy, Eric Boerwinkle, Grace Kubin, Stephen J Pont, Jennifer A Shuford

**Affiliations:** Texas Department of State Health Services, Austin, Texas; Texas Department of State Health Services, Austin, Texas; Texas Department of State Health Services, Austin, Texas; The University of Texas Health Science Center, Houston, Texas; UTHealth Science Center at Houston - School of Public Health, Houston, Texas; UTH, Houston, Texas; Texas Department of State Health Services, Austin, Texas; Texas Department of State Health Services, Austin, Texas; Texas Department of State Health Services, Austin, Texas

## Abstract

**Background:**

Texas is the second largest state in the country and has significant geographic and demographic diversity placing it at the forefront for identifying and responding to emerging infectious diseases. Therefore, it is critical to establish pathogen surveillance programs with statewide reach and local impact to meet public health needs. The Texas Department of State Health Services (DSHS) partnered with the University of Texas Houston School of Public Health (UTSPH) to create a statewide SARS-CoV-2 variant surveillance monitoring network project (the Network). This Network can be a model for monitoring other emerging pathogens in Texas.

**Methods:**

A network was formed among DSHS, six academic centers, two commercial laboratories, and one public health laboratory for high throughput SARS-CoV-2 sample procurement, sequencing, and analysis for Texas. The program developed sample procurement and sequencing capacity across the state and electronic genomic data reporting to DSHS. Partners were selected based on expertise and area of geographic need. Protocols were established to meet high data quality and reporting standards. Working committees and shared data visualization tools were established to ensure collaboration among partners. The steering committee and workgroups were established to support network activities and enable DSHS to monitor epidemiological dynamics and transmissibility and respond to SARS-CoV-2 variants across each public health region.

**Results:**

The Network has successfully established a state-wide system for sample collection, whole genome sequencing, reporting and analysis of data. At a regular cadence, the six academic centers acquire SARS-CoV-2 samples, sequence them in their respective labs and report the corresponding sequence or test data to DSHS and UTSPH. As of April 21, 2023, a total of 17,257 samples have been collected (Figure 1) and a total of 14,080 samples have been sequenced (Figure 2).

**Figure:**
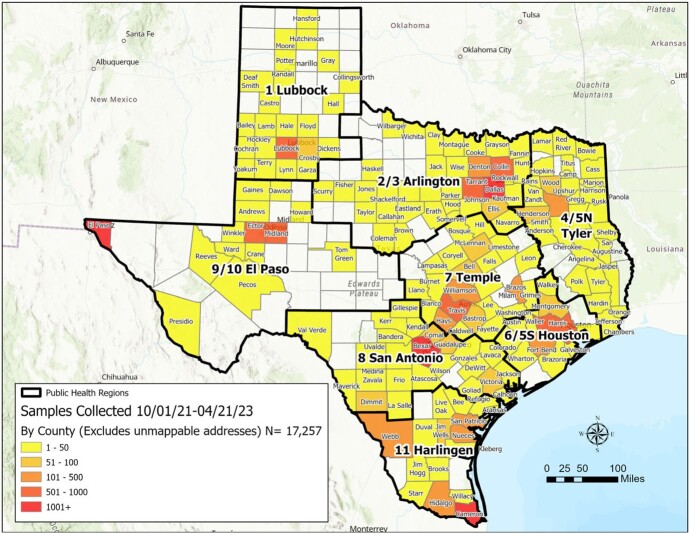

**Figure:**
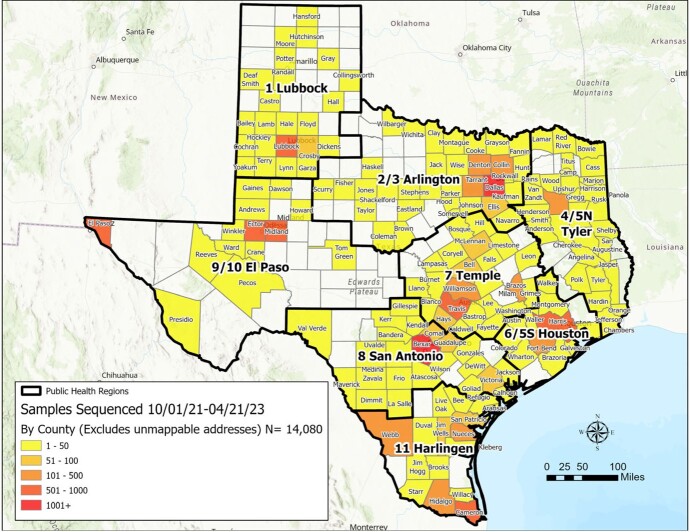

**Conclusion:**

The network of public, academic and private partners has effectively met the need for SARS-CoV-2 sequence surveillance in this large and diverse state. With this established infrastructure in place, expanding to study other emerging pathogens in Texas is feasible.

**Disclosures:**

**Saroj Rai, PhD, MPH**, Novartis: retirement in form of stock|Novartis: Stocks/Bonds

